# A review of risk concepts and models for predicting the risk of primary stroke

**DOI:** 10.3389/fninf.2022.883762

**Published:** 2022-11-15

**Authors:** Elizabeth Hunter, John D. Kelleher

**Affiliations:** ^1^PRECISE4Q Predictive Modelling in Stroke, Technological University Dublin, Dublin, Ireland; ^2^ADAPT Research Centre, Technological University Dublin, Dublin, Ireland

**Keywords:** stroke, predictive modeling, machine learning, risk, epidemiology

## Abstract

Predicting an individual's risk of primary stroke is an important tool that can help to lower the burden of stroke for both the individual and society. There are a number of risk models and risk scores in existence but no review or classification designed to help the reader better understand how models differ and the reasoning behind these differences. In this paper we review the existing literature on primary stroke risk prediction models. From our literature review we identify key similarities and differences in the existing models. We find that models can differ in a number of ways, including the event type, the type of analysis, the model type and the time horizon. Based on these similarities and differences we have created a set of questions and a system to help answer those questions that modelers and readers alike can use to help classify and better understand the existing models as well as help to make necessary decisions when creating a new model.

## 1. Introduction

Based on the most recent Global Burden of Disease estimates in 2019, stroke is the second leading cause of death worldwide and the third leading cause of death and disability. As of 2019, it was estimated that global cost of stroke was approximately 1.12% of the global GDP or over 891 billion US dollars. Globally the burden of stroke is increasing: there was a 70% increase in incident strokes and a 43% increase in stroke deaths between 1990 and 2019 (Feigin et al., 2022).

Prevention strategies tend to fall into two main categories,“high-risk” strategies that target individuals who have been identified as having a higher than average risk for stroke and population strategies that aim to reduce risk factors within the population (Rose, 2001). Thus, to reduce the burden of stroke on society it is essential to understand the risk factors associated with primary stroke and to identify those who are at risk. We consider a primary stroke to be the first stroke that an individual has. One way that this can be done is through using statistical or machine learning models. There are a number of models in existence that are used in different capacities to estimate an individual's stroke risk or the contribution of risk factors to stroke risk. However, the models differ in a number of ways, from the type of risk that is being predicted, to the model being used. These differences can lead to differences in the way that the risks produced by the model should be interpreted. Therefore, it is important to understand the different characteristics of the models used in predicting primary stroke risk. Additionally, in the context of stroke prevention it is important to understand what risk is telling us and what questions the concept or risk is primarily used to ask. Although there are a number of systematic and other reviews on primary stroke risk prediction, Lloyd-Jones (2010), Siontis et al. (2012), Jeena and SukeshKumar (2018), and Xu et al. (2021) these reviews focus either on describing individual models, or comparing the predictive abilities or bias of existing models. Thus, there is a gap in the literature for review that not only describes modeling methods but aims to help readers to better understand the different characteristics of risk models and how they should be interpreted. With this paper we aim to fill that gap.

Many of the risk factors of stroke are similar to risk factors for other cardiovascular diseases (CVD) and as such in some models stroke is not differentiated from other CVD events. Thus, before modeling stroke risk, any study must first decide on the answer to the following question:

1. Is the model for stroke specific risk or general CVD risk?

Once the answer to this question is determined, the study then needs to determine the type of risk being modeled by answering the following:

2. Is the study predicting stroke/CVD or looking at risk factors?

The answer to this question determines what other questions the study can ask. (a) If the study is predicting stroke/CVD, we see the following questions as potentially the focus of a study:

i. What is the probability (or risk) of an individual having a stroke/CVD within a preset time window (e.g., the next 10 years)?
ii. What is the probability (or risk) of an individual having a stroke/CVD within their lifetime?
iii. Within a given time window when is a person likely to have a stroke/CVD?

Whereas, if the answer to question 2 is instead (b) the study aims to look at risk factors, we see the following as potential questions:

i. What are the factors that have a strong association with stroke/CVD (i.e., what are the risk factors of stroke/CVD)?
ii. Do different factors have a stronger association with a certain type of stroke/CVD (ischemic vs hemorrhagic)?
iii. Are there risk factors that are more important for individuals with a co-morbidity?

These questions, however, are not answered by all models, and study design can have an impact on the type of model chosen. The answers to these questions can be expressed in different ways, for example as absolute risk, relative risk, odds ratios, and hazard functions. In this paper we:

- Set out the different factors that should be considered in modeling stroke risk.
- Based on a review of the current guidelines and research literature on modeling stroke risk, provide:
  - A set of questions that researcher can use to help define their model.
  - A classification that can help select the appropriate model type for different scenarios.

The paper proceeds as follows: the next section starts by describing the different types of risk and their advantages and disadvantages. Section 3 then describes the two main classes of models used to predict stroke risk. The following sections discuss the different ways that we classify the models looking at the time horizon of the model (Section 4), the event type (Section 5) and identifying risk factors (Section 6). Finally, in Section 7 we analyze the inter dependencies of the risk type, the model type, the time horizon and the event type.

## 2. Risk

**Figure 1:**
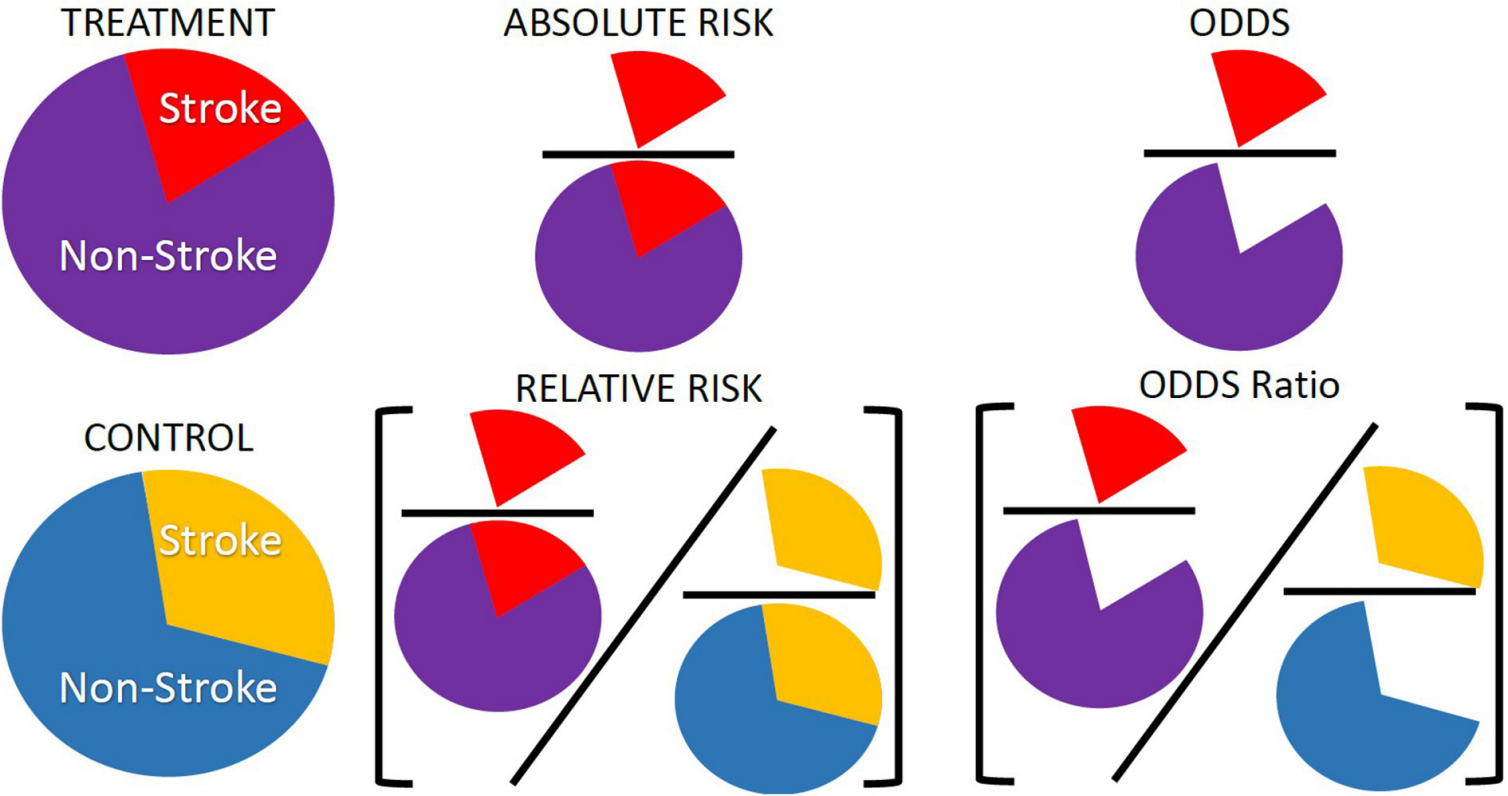
Representation of absolute risk, relative risk, and odds ratios. These are three of the common risk or association types presented in modeling studies and are often used interchangeably or misinterpreted when there are distinct differences between them. Absolute risk is often presented as a percentage and is the number of individuals with an event, number of strokes, over the total number of individuals in a group, number of strokes and non-strokes. Relative risk is a ratio of absolute risks, often treatment over control. Odds ratios are a measure of association rather than a risk. Odds are the number of times an event occurred in a group, number of strokes, over the number of times the event did not occur, number of non-strokes. Odds ratios are a ratio of two odds, often the ratio of the odds in a treatment group over the ratio of odds in a control group.

Although risk is a concept used in our daily lives, it is a concept that is often ill understood or misinterpreted (Malenka et al., 1993). Risk can be thought of as either the probability of an event occurring or a combination of that probability and the severity of the event. In the models presented here we do not consider the severity of the event and thus take risk to be the probability of the event occurring. There are, however, more than one way to present risk: it can be presented as an absolute risk or a relative risk. Beyond pure measures of risk, measures of association such as odds ratios or hazard ratios are often used when risk cannot be calculated directly. In any discussion on risk and risk models it is important to understand the different types of risk that are used in relation to health care, how these risks should be interpreted and presented along with potential ways the risks can be misinterpreted. Figure 1 shows representations of the different kinds of risks and associations discussed in the paper. In the following sections we first discuss the difference between absolute and relative risk, then discuss measures of association and finally the types of studies and data that might influence what type of risk or association is presented.

### 2.1. Absolute and relative risk

Risk can be either absolute or relative. Absolute risk is a probability and can be calculated as the number of events in a given group divided by the total population of that group. The group considered is defined by the study. It can be all patients in a study, it can be all patients with a given risk factor, or all patients with similar characteristics or a combinations of characteristics such as age and sex. It is often presented as a percentage. For example, an individual would have a x% risk of having a stroke in a given time period, which can also be interpreted as out of 100 individuals with similar risk factors to you, x of them will have a stroke in the time period (Thomson et al., 2005).

An alternative to looking at absolute risk is to look at relative risk. Relative risk is the ratio of the absolute risk of two groups. For example, the ratio of the risk of a treatment and a control group. Relative risk is used more often in medical literature, the press, and clinical encounters compared to absolute risk (Malenka et al., 1993). Relative risk is also presented as a percentage but will be often related to change in risk factors, for example a relative risk statement would be that a patient would reduce their risk of having a stroke by x% if their blood pressure was reduced (Thomson et al., 2005). As the relative risk is a ratio of two absolute risks, if the relative risk and one of the absolute risks is known the second absolute risk can be found (Malenka et al., 1993). For example, if the relative risk of a stroke for those on a blood pressure medication compared to a control group is known and the absolute risk of a stroke for the control group is known, multiplying the relative risk by the absolute risk of the control group will give the absolute risk of the treatment group.

Although relative risk is used more often, many feel that absolute risk is the most meaningful form of risk when considering clinical decision making (Malenka et al., 1993) and should be used over relative risk (Thomson et al., 2005). Absolute risk is also less open to misinterpretation than relative risk because it is not a comparison between two groups. To fully understand the risk of the treatment group in relative risk the risk of the control group also needs to be known whereas absolute risk gives the direct risk of the treatment group (Paling, 2003) and if competing risks^1^ are a factor absolute risk is more appropriate to consider then relative risk (Benichou and Gail, 1990). However, there are some disadvantages of using absolute risk, as it is likely to be presented with a smaller percentage than relative risk, both patients and physicians when presented with an absolute risk and a relative risk tend to choose the treatment that is associated with the relative risk (Malenka et al., 1993; Thomson et al., 2005).

### 2.2. Measures of association

Sometimes there is a reason why absolute or relative risk cannot be directly calculated and a measure of association is calculated instead. This is often due to the types of study the data used to calculate risk has come from. If a cohort study is used then measures of association should be used over pure measures of risk. This is discussed further in the next section. The most common types of measures of association are odds ratios or hazard ratios. Odds are relative probabilities and are expressed as the ratio of the probability that an event will happen to the probability that it will not happen, and odds ratios are the ratio between the odds of the treated group and the odds of the control group. They can be interpreted as whether someone with a risk factor is more or less likely to experience the outcome of interest compared to someone without the risk factor (Norton et al., 2018). Odds ratios are a way to look at the size of an effect a treatment or a factor can have on an outcome (Schechtman, 2002). Although not by strict definition a measure of risk, the odds ratios are related to the relative risk and thus the absolute risk. With the following formula often used to convert from odds ratios to relative risk:


(1) $$\begin{array}{l} RR=\frac{OR}{\left(\left(OR-1\right)*P_{0}\right)+1} \end{array}$$

Where RR is relative risk, OR is odds ratios and *P*_0_ is the prevalence of stroke in the control group (Shrier and Steele, 2006). One of the disadvantages of odds ratios is that they are hard to interpret and are often misinterpreted as relative risk. If the event is rare than the odds ratio will approximate the relative risk, however, when the event is common the odds ratio is not a good approximation of relative risk (Cummings, 2009). This can be seen when looking at Equation 1. If the event is rare the prevalence in the control group, *P*_0_, will be close to 0 thus the denominator in the equation will tend to 1 leaving the relative risk equal to the odds ratio.

A hazard ratio is a term used in survival analysis where a hazard is the probability of an event occurring within a given time frame. A hazard ratio describes the relationship between the event and survival time and is generally defined as the ratio of the hazard for one individual or group to another individual or group (Kleinbaum and Klein, 2012). Hazard ratios are dependent on the time period and can change over time. The time period is defined by the study and can be short term over a few years or long term over a lifetime. While they are often interpreted as relative risk ratios, this is not entirely true and can be misleading unless the comparison is being made over small time intervals (Stare and Maucort-Boulch, 2016). Despite their limitations hazard ratios are useful in understanding time to event data (Sutradhar and Austin, 2018). Similar to odds ratios if some additional information is known other risk measures can be calculated with a hazard ratio. Hazard ratios can be adjusted to find the absolute risk (Austin, 2010). If the death rate or the rate of the disease occurrence for the control group is known then the relative risk can be calculated from the hazard ratio (Shrier and Steele, 2006) and then converted to absolute risk.

### 2.3. Types of studies and data

As we have seen risk can be represented and calculated in a number of ways, and the choice of risk calculation can be influenced by the available data and the study. The data and type of study can determine whether a measure of risk (absolute or relative) is used or a measure of association (odds or hazard ratios). There are two types of studies used to estimate risk directly from the data, cohort studies and case-control studies. Cohort studies start with a population typically free from a given disease and track the individuals forward through time to see if they develop the disease, while case-control studies look at a group of individuals with the disease and a group of individuals without the disease and look at their risk factors going backwards in time.

If calculating risk directly from data we calculate risk in relation to a specific risk factor. For example, the risk of having a stroke if a patient has diabetes. When calculating directly from data, both absolute and relative risk calculations are only meaningful if used on cohort studies, and cannot be directly estimated from case-control studies because a case-control study starts with two sample populations, one that already has the condition and one that does not and looks backwards to see exposures. Thus, the prevalence of the diseases in the full sample of the case-control study is predetermined by study design and in order to calculate relative or absolute risk the actual prevalence of the disease or event in the population needs to be known. By contrast odds ratios can be used in both cohort and case-control studies as they compare the rates of the event in one population to another and the actual prevalence of the disease is not needed to calculate odds ratios, but they are not typically used for cohort studies as risk can be calculated directly in these studies (Schechtman, 2002).

As an alternative to directly getting risk or association measures from the data source, modeling methods can be used to determine the contribution of risk for each risk factor and can predict an individual's risk based on the combinations of these risk factors. Typically modeling is done using data from cohort studies. However, although it is standard when calculating risk directly from a cohort study to calculate absolute or relative risk, in modeling odds ratios or hazard ratios are found. These measures of association can then be converted to absolute or relative risk if desired (Schechtman, 2002). Modeling allows us to not only look at the risk of a cohort or a group in the data but allows us to predict the risk of an individual not in the cohort or to predict how someone's risk might change if their risk factors change. Furthermore, while direct calculations of risk often only focus on one risk factor, modeling can take multiple risk factors into account and can help separate the contribution of each risk factor to an individual's risk.

## 3. Risk models

There are a number of methods used to model stroke risk for primary prevention. The type of model used can be influenced by a number of factors including the data being used, the time horizon of the model, and the type of risk being modeled. Understanding the model type and how it estimates risk is important in understanding how to interpret the results of the model. The main types of models used to predict stroke risk that have been adopted by clinical guidelines are survival analysis and regression, in particular logistic regression (Lloyd-Jones, 2010; Jeena and SukeshKumar, 2018) thus we limit the discussion in the following sections to these two methods. There are, however, studies that use other models, in recent years there has been an increase in the number of papers using machine learning models to predict stroke risk. For example, studies have used decision trees, support vector machines (SVM), random forest, and naive bayes (Wolfson et al., 2015; Wongvibulsin et al., 2019; Jamthikar et al., 2020; Soto-Cámara et al., 2020). However, current stroke risk prediction guidelines use either logistic regression or survival analysis (Chun et al., 2021). Additionally, most machine learning articles published recently are comparative in nature, showing the improvement in the performance of different machine learning techniques over the more traditional methods or showing which machine learning technique gives the best performance (Li et al., 2019; Shoily et al., 2019; Chun et al., 2021; Dritsas and Trigka, 2022; Lip et al., 2022) This is an important step in improving the field, however, such papers do not focus on risk concepts. In fact, most recent machine learning risk prediction papers do not mention the type of risk that is calculated (relative, absolute, hazard ratio, odds ratios etc.) (Li et al., 2019; Shoily et al., 2019; Chun et al., 2021; Dritsas and Trigka, 2022; Lip et al., 2022). Thus, while understanding these newer methods and how they might improve upon more traditional methods is useful, we do not include machine learning models in our review of risk concepts as we are unable to determine what type of risk the models are predicting from most articles, and focus on more traditional regression and survival analysis models.

Both regression techniques and survival analysis can be considered propensity models, models that predict outcomes in the future based off of a set of descriptive features. Propensity models consider two time periods, the observation period and the outcome period. The observation period is the time when the descriptive features are determined and the outcome period is when the response variable is determined (Kelleher et al., 2015). For the case of both logistic regression and survival analysis for stroke risk prediction, the outcome period world be the time period when the risk factors are collected for the patient. The observation period is the time period when the patient is followed up with to determine if they have had a stroke or not.

### 3.1. Regression

Regression models are a family of statistical models that are used to estimate relationships between independent and dependent variables and are considered error-based learning within machine learning as the model parameters are fitted by minimizing total errors (Kelleher et al., 2015). Linear regression is the regression technique most commonly thought of but there are a number of other regression techniques, commonly referred to as generalized linear models, that are used in primary stroke risk predictions. Robbins et al. (2002) discusses using binomial regression, while McNutt et al. (2003) discuss the use of log-binomial and Poisson regression. However, the type of regression model most typically used for predicting stroke risk is logistic regression, thus in the following section we discuss logistic regression in more detail.

#### 3.1.1. Logistic regression

Logistic regression models are a type of regression model that has been adjusted to predict categorical response variables (Kelleher et al., 2015). Although versions of logistic regression exist to deal with ordinal categorical variables with more than two outcomes, the main focus of logistic regression is modeling dichotomous variables which makes it a natural choice for predicting stroke risk as the predicted explanatory variable would be stroke or no stroke during the time horizon studied (i.e., the observation period). The equation for a logistic regression model is:


(2) $$\begin{array}{l} p=\frac{1}{1+e^{-\left(\beta_{0}+\beta_{i}x_{i}\right)}} \end{array}$$

which can be rewritten in the following form:


(3) $$\begin{array}{l} \log\frac{p}{1-p}=\beta_{0}+\beta_{i}x_{i} \end{array}$$

Where p is the probability of the event occurring within the observation period, $\log\frac{p}{1-p}$ are the log odds of the event occurring within the observation period, β_*i*_ are the coefficients, *x*_*i*_ are the independent variables and β_0_ is the intercept of the model and the resulting log odds if there are no independent variables in the model. As the model predicts log odds instead of probabilities, the results and coefficients of a logistic model are not directly interpretable. To interpret the log odds the coefficients need to be exponentiated and then the resulting odds ratios can be converted to probabilities (Norton et al., 2018). Thus, using a logistic model does not provide a technical measure of risk but rather odds ratios. As discussed in previous sections if the incidence in the control group is known the odds ratios can be converted to relative risk. The timing of the event in question, is only taken into account in the sense of if the event occurred within a time window. Thus, logistic regression can be used to answer question 2 (a) i. and 2 (a) ii. from the introduction: what is the probability of an individual having a stroke or CVD event in a given time window, and what is the probability of an individual having a stroke or CVD event within their lifetime? Similarly, logistic regression can be a tool to use when the exact time to the event is not known. If the only available information is that an individual has a stroke within the time window and not the time to the stroke, logistic regression might be a better option than survival analysis. Additionally, looking at the β_*i*_ coefficients can provide information on the odds ratios for individual independent variables or predictors and can be used to answer questions looking at risk factors: 2 (b) i what are the risk factors for stroke and CVD, 2 (b) ii. do risk factors differ between stroke type and CVD and 2 (b) iii. do risk factors differ with comorbidities, from the introduction.

A number of models to predict stroke risk or to determine different risk factors for primary stroke use logistic regression. Although they later changed their technique to use Cox Hazard models, the early stroke risk models from the Framingham Heart Study used logistic regression to determine the probability of having a CVD event in 10 years (Kannel et al., 1976). Logistic regression was also used in the EUROSTROKE project to analyze the effects of different risk factors for both ischemic and hemorrhagic stroke on three different European cohorts (Bots et al., 2002b,c). Also, in order to determine the risk of ischemic stroke for women who have had preeclampsia, Brown et al. (2006) use a logistic regression model to look at the odds ratios of the different risk factors.

### 3.2. Survival analysis

Survival analysis is a term used to describe a number of statistical methods where the variable of interest is the time to an event occurring. Thus, it is sometimes also referred to as time to event analysis. There are a wide range of different survival analysis methods used such as Kaplan-Meier Survival Curves, Log-Rank test, Cox Regression and the Weibull model (Kleinbaum and Klein, 2012). Survival analysis often considers two main functions, the survivor function, the probability that a person survives longer than a given time, and the hazard function, the potential per unit of time for the event in question to occur given a person has survived up to a given time point. As the hazard function focuses on the event occurrence, while the survival function focuses on the event not occurring, they can be seen as providing opposite sides of the same information and if one function is known the other can be derived. When running a survival regression model, such as a Cox or Weibull regression model, only the hazard function is used in the model. Survival analysis can be done to examine the relationships between independent variables and survival time, to compare survivor and hazard functions, and to estimate these functions from existing survival data. Survival analysis has some advantages over other methods: it takes into account not just when an event occurred but the time to the event occurrence as well. Survival analysis can therefore answer question 2 (a) iii. from the introduction about when a person is likely to have a stroke within a time window. Survival analysis is also better at handling censored data^2^ (Kleinbaum and Klein, 2012). Although the output of a survival analysis model is typically in the form of hazard ratios, the hazard ratios can be converted to relative risk as discussed previously.

The most common survival analysis method used is Cox regression which we discuss in more detail in the following section. We also briefly discuss the Weibull model as it is used in the SCORE model which is recommended under the European guidelines for cardiovascular prevention (Piepoli et al., 2016).

### 3.2.1. Cox regression models

The Cox proportional hazard model is a survival analysis technique that assumes that the hazard at a given time is equal to the product of the baseline hazard function, *h*_0_(*t*), and the exponential of the sum of the independent variables, $e^{\Sigma \left(\beta_{i}X_{i}\right)}$. Where *t* is time, *X*_*i*_ are the independent variables that would be features included in the model such as sex or a diagnosis of diabetes, and β_*i*_ are the coefficients for each independent variable. The baseline hazard function is the hazard function that would be left if there were no features included in the model thus the hazard function can be seen as similar to the intercept, β_0_, in the logistic regression model. As the baseline hazard function is dependent on time, the product of the baseline hazard function and the exponential of the sum of the independent variables gives us a hazard function *h*(*t, X*) that shows how an individual's hazard changes over time given a set of features (Xs) that are not time dependent. The equation for a Cox Proportional hazard model is:


$$h\left(t,X\right)=h_{0}\left(t\right)e^{\Sigma \left(\beta_{i}X_{i}\right)}$$

The Cox model is considered semiparametric because the baseline hazard function is unspecified, this means that the survival function is also unspecified. The Cox model is robust at estimating the baseline hazard, with the results approximating those of the correct parametric model thus it can be used when the information about the baseline hazard is unknown (Kleinbaum and Klein, 2012). The Cox proportional hazard model does have an important assumption of proportional hazards this means that the hazard ratios are constant over time (Xue et al., 2013). If this assumption does not hold for all of the independent variables, a stratified Cox model can be used instead that controls for the independent variables whose hazards are not proportional by stratifying them into a number of different groups and creating separate hazard functions for each of the stratified groups. The equation for a stratified Cox model is given below where g represents the different strata. For example, if it was determined that the hazards were not proportional for males and females, there would be a two strata with a separate baseline hazard function for males and a separate baseline hazard function for females (Kleinbaum and Klein, 2012).


$$h_{g}\left(t,X\right)=h_{0_{g}}\left(t\right)e^{\Sigma \left(\beta_{i}X_{i}\right)}$$

In more recent iterations, the Framingham stroke and CVD risk models have used Cox proportional hazard models (D'Agostino et al., 2008; Dufouil et al., 2017). To predict the separate risks for coronary heart disease, ischemic stroke and hemorrhagic strokes (Zhang et al., 2005) use Cox models. Jee et al. (2008) use the Cox proportional hazard model to study stroke risk prediction using a Korean cohort study, Veronesi et al. (2013) use Cox models to make long term predictions of major coronary events in a Southern European population, and Banerjee et al. (2012) use Cox models to estimate the risk of ischemic stroke in a population with diabetes.

### 3.2.2. Weibull model

Another model sometimes used in survival analysis is the Weibull model. The Weibull model has the same underlying structure as the Cox model, where the hazard function is equal to the product of a baseline hazard function and the exponential sum of the independent variables. However, unlike the Cox model the Weibull model is a parametric model where the baseline hazard is specified as λ*pt*^*p*−1^. Although not included in the model, the corresponding baseline survival function for the Weibull baseline hazard function is *e*^−λ^*t*^^*p*^. The equation for the Weibull model is:^


$$h\left(t,X\right)=\lambda pt^{p-1}e^{\Sigma \left(\beta_{i}X_{i}\right)}$$

Where *t* is time, λ is a constant hazard, and *p* is referred to as the shape parameter: if *p* > 1 then the hazard will increase as time increases, if *p* < 1 the hazard will decrease with time and if *p* = 1 the hazard is constant. Similar to the Cox model the Weibull model also assumes proportional hazards. As the Cox model will approximate the actual baseline hazard when it is not known, running a Cox model on the same data is sometimes used to validate the use of a Weibull model (Conroy et al., 2003).

The Weibull model is used to create the SCORE risk model to estimate the risk of fatal cardiovascular disease in Europe (Conroy et al., 2003) that is presented in the European guidelines on cardiovascular disease prevention (Piepoli et al., 2016). A Weibull model is also used in Assmann et al. (2007) to predict the risk of coronary heart disease although they also use a Cox Proportional hazard model for stroke risk prediction.

## 4. Time

There are two aspects of time that distinguish between model types and risk scores. The first aspect we discuss is the time horizon included in the risk score and the second is if the time to the event is considered in the model or not.

### 4.1. Time horizon

One of the important factors defining a risk score is the time period over which the risk applies. While short term (10 years) risk was initially the time horizon suggested in the European guidelines on cardiovascular disease prevention (De Backer et al., 2004), in recent years the guidelines have changed to also give consideration to lifetime risk. The update to the guidelines was motivated by the consideration that short term risk might ignore the risks in younger individuals and women (Piepoli et al., 2016). Seshadri et al. (2006) show how the stroke risk of an individual can change when considering different time frames. They examine at short term (10 year), intermediate term (20 and 30 years), and lifetime risk and find that when risk is calculated at 55 or 65 women have a higher lifetime risk than men, conversely the 10 year risk for women at age 55 or 65 is lower than the 10 year risk for men. While short term risk factors for cardiovascular disease are well known, factors that increase long term and lifetime risk are less predictable and have not been studied as frequently (Lloyd-Jones et al., 2006). Although there are some models that calculate the intermediate term stroke risk, the main focus in the literature and the current guidelines on stroke prevention is on short term and lifetime risk. In the following sections we discuss short term and lifetime risk in more detail.

#### 4.1.1. Short term risk

Short term risk, usually defined as 10 years, is commonly measured when predicting initial stroke risk. It is the time period that is used in the model for cardiovascular risk presented in the European Guidelines on cardiovascular disease prevention in clinical practice (Piepoli et al., 2016) and is used in the Framingham stroke models (Kannel et al., 1976; Wolf et al., 1991; D'Agostino et al., 2008), and the European SCORE project (Conroy et al., 2003). Other models have been developed to predict even shorter term risk of 5–7 years (Lumley et al., 2002). The follow up period for a study involving short term risk of stroke only requires a length of follow up to as least as long as the time horizon being considered. This potentially reduces the need to account for right censoring as compared with a longer term risk because of individuals dropping out from the study. Modeling short term risk has been done with a number of different methods including survival analysis and regression (Kannel et al., 1976; Wolf et al., 1991; Lumley et al., 2002; Conroy et al., 2003).

Short term risk can change considerably as an individual ages. Seshadri et al. (2006) show how an individual's risk changes and how short term risk for a stroke can change as an individual ages along with how the patterns of short term risk can change between groups. When calculated for individuals aged 65, the 10-year risk of stroke is higher for men than women; when calculated for individuals at age 75 the risk for men and women were equal, and when calculated for individuals aged 85 the risk for women was higher than men. This might mean that in a short term stroke risk model age might need to be considered in a different way to a long term risk model as all risk factors might not be proportional with changes in age. To account for this change in short term risk by age and for the non-proportionality in the risk factor contribution to stroke risk by age (Hunter and Kelleher, 2022) create a set of age specific logistic regression models where age is not included as a factor but independent models were created for four different age groups.

Although short term risk of stroke is often used and modeled, it is expected to be low in certain groups and can potentially result in lack of necessary early interventions for younger individuals and in particular women. Thus, recently there has been a push to consider both short term and lifetime risk in order to fully understand an individual's risk of stroke (Piepoli et al., 2016).

#### 4.1.2. Lifetime risk

Lifetime risk is a risk presented in absolute terms and therefore may lead to better interpretation by a clinician. It can also help provide a better idea of the burden of disease on society and on the individual (Seshadri et al., 2006) and provides the answer to the question 2 (a) ii. in the introduction of how likely an individual is to have the event occur within their lifetime. This can be vastly different from the risk of the event in the short term. While a younger individual might have very low short term risk, their lifetime risk may be high due to the presence of only a single risk factor that overtime will lead to higher stroke risk. Presenting lifetime risk of breast cancer has lead to an increase in early screenings and thus lowered the population burden of the disease (Lloyd-Jones et al., 2006). In calculating lifetime risk it has been found that individuals can be classified into short and lifetime risk using a stepwise risk model by first calculating risk for the short term (10 years) and lifetime, and then classifying individuals into three categories: low short term and low lifetime risk, low short term and high lifetime risk, and high short term risk (Lloyd-Jones et al., 2004; Lloyd-Jones et al., 2006; Marma et al., 2010). A study by Marma et al. (2010) find that two thirds of US adults with low short term risk have high lifetime predicted risk, showing the importance of looking at lifetime risk to reduce the overall burden of stroke. Similarly, Lloyd-Jones et al. (2006) find that the presence of a single risk factor at the age of 50 is associated with high lifetime risk.

Lifetime risk is typically calculated using time to event or survival analysis. When predicting lifetime risk there are additional factors that are not always considered for short term risk. To predict lifetime risk a longitudinal study that continues for a long enough period of time so that the majority of the individuals in the study have reached an age where we can consider them to have “survived” without a stroke is necessary. Competing risks are also important when calculating lifetime risk as the risk factors that might lead to an individual having a high lifetime risk of stroke might also result in a high lifetime risk for other diseases. Thus, right censoring becomes a more important consideration as those who have dropped out of a study due to death may have been at high risk for a stroke but have died due to another condition (Lloyd-Jones et al., 2006). Lifetime or long term risk is often determined for not just stroke, but rather groups stroke along with other cardiovascular diseases (Lloyd-Jones et al., 2004; Lloyd-Jones et al., 2006; Marma et al., 2010; Wilkins et al., 2012; Veronesi et al., 2013).

### 4.2. Time to event

The handling of the observation period, the time period when the patient is followed up with to determine if they have had a stroke or not, is one place where survival analysis and regression differ. While both methods look at a given time period, for example 10 years, to determine if patients had a stroke or not, regression models do not consider when in that time period an individual has had a stroke. The resulting risk from the regression model results will be the same regardless of whether the stroke was 1 year from the observation period or 10 years. Survival analysis, however, considers the time to the event in the risk calculation. A stroke 1 year from the observation period would result in higher hazard ratios than a stroke 9 years from the observation period. Thus, if it is important to consider the time to the event (as it might be in a lifetime risk model), survival analysis would be the better methodology.

## 5. Prediction

As outlined in the introduction, when creating a model for stroke risk prediction there are two main types of models, one that predicts the event or stroke, and the other that analyzes risk factors. Although the models to predict stroke risk can differ on the type of risk they are predicting, they can also differ in the event the model is predicting. Some models aim to predict the risk of a specific type of stroke, while others predict risk of stroke more generally, and still others predict the risk of any cardiovascular event. The following sections discuss the models that predict stroke or cardiovascular risk in more detail, specifically focusing on the type of event predicted. The models discussed in the following sections potentially answer questions 2 (a) i. what is the probability of having a stroke or cardiovascular event in a given time window, 2 (a) ii. what is the probability of having a stroke or cardiovascular event in ones lifetime or 2 (a) iii. when in a time window will someone have a stroke or cardiovascular event.

### 5.1. Cardiovascular disease

As the risk factors for stroke are similar to the risk factors for other cardiovascular disease, often when creating a risk model the event type is cardiovascular disease and not specifically stroke. The SCORE project (Conroy et al., 2003), used in the European Guidelines on cardiovascular disease prevention in clinical practice (Piepoli et al., 2016) to predict fatal cardiovascular disease, does not differentiate between the types of cardiovascular disease. The SCORE model replaced the risk chart for coronary risk profile previously in the European guidelines created from the Framingham heart study (Anderson et al., 1991). A general coronary event model was used for calculating a risk score on the Prospective Cardiovascular Munster (PROCAM) study, in the CUORE cohorts project (Ferrario et al., 2005), and in additional Framingham heart study risk profiles (Wilson et al., 1998; D'Agostino et al., 2008).

As those at risk for stroke are also at risk for other cardiovascular events, in the long term it may be more useful to understand a patient's overall risk for CVD. Indeed, the majority of lifetime or long-term risk models predict cardiovascular disease risk instead of specifically stroke risk to account for the competing risk overtime (Lloyd-Jones et al., 2004; Lloyd-Jones et al., 2006; Marma et al., 2010; Wilkins et al., 2012; Veronesi et al., 2013). Additionally, in some cases data restrictions prevent the calculation of stroke risk specifically. Some longitudinal population cohort studies, such as the Irish Longitudinal Study on Aging (TILDA), only collect information if a patient has had a CVD event and not the specific event (TILDA, 2016).

These models have the disadvantage of not being made to specifically predict stroke and take a more general approach to cardiovascular disease. Although the risk factors for stroke and other CVD are similar, Zhang et al. (2005) show that when creating separate models by stroke type and coronary heart disease (CHD), the contributions of the risk factors vary between models and not all risk factors significantly contribute to each model. For example, they include BMI in their CHD model but in neither of their stroke models. Thus, if a patient is focused on their risk of stroke, a general CVD model may not provide all of the necessary risk factors, or may cause an individual to focus on a risk factor that does not have as much of an effect on lowering their stroke risk.

However, as the risk of stroke is strongly linked with the risk of other cardiovascular diseases, often the risk scores that include both stroke and other cardiovascular diseases are more clinically useful (Boehme et al., 2017). As many types of heart disease are a risk factor for stroke and stroke is a risk factor for coronary heart disease (American Stroke Association, 2020), reducing a patients overall risk for cardiovascular disease can help to reduce the overall incidence of stroke.

### 5.2. Stroke

A model specifically designed to predict the risk of stroke of an individual can help in the reducing the risk of stroke for patients with high non-modifiable factors, such as genetics or family history of stroke. There is evidence family history of stroke significantly increases the risk for stroke, this could be due to a number of factors such as shared family exposure, genetic disorders and other gene variants (Boehme et al., 2017). However, these factors are non-modifiable and those with a high non-modifiable risk need to focus on their modifiable risk factors to reduce their overall risk of a stroke. Thus, a stroke specific model may help these individuals more than a general CVD model as it might identify modifiable factors that specifically increase stroke risk that the patient who is genetically predisposed to a stroke might be able to change.

A number of models have been created to model the risk of stroke. These models typically use data from studies designed to look at population risk factors for CVD events over time. For example, data from the Framingham heart study had been used to predict the risk of stroke (Wolf et al., 1991; Seshadri et al., 2006), similarly data from the Cardiovascular Health Study has been to create a stroke risk model (Lumley et al., 2002). Although some of the models for stroke look at lifetime risk (Seshadri et al., 2006) and short term 5-year risk (Lumley et al., 2002), most of the models predict 10 year risk (Wolf et al., 1991; Jee et al., 2008; Chien et al., 2010). The emphasis on 10 year risk is likely due to the guidelines for stroke and cardiovascular prevention such as the European guidelines that focus on 10 year risk (De Backer et al., 2004). Additionally, a shorter term risk model allows for less competing risks from other cardiovascular diseases or other conditions. These models however, do not differentiate between types of stroke and focus on an individual's overall stroke risk even though an individual might have factors that might make them more or less susceptible to a specific type of stroke.

Often risk models that are specifically for those with ischemic stroke are created for populations that already have a given risk factor that is known to increase the risk of ischemic stroke. One such factor that many models are created for is atrial fibrillation with the CHADS2 score widely used to predict ischemic stroke in atrial fibrillation patients (Gage et al., 2001), along with a number of other models that have been created using a population with atrial fibrillation (Singer et al., 2013; Kang et al., 2017).

## 6. Identifying risk factors

As an alternative to predicting stroke risk, models can also be used to better understand different risk factors for a stroke. These models would not necessarily be used to produce an overall risk of a stroke, but could be used to identify if there are certain risk factors that contribute more to stroke risk than others, or if there are risk factors that are more important in subsets of the population. Thus, the important results from these models would be the odds ratios or hazard ratios associated with each risk factor. The following sections discuss models that look at the difference in risk factor by stroke type (ischemic or hemorrhagic) and then risk factors for stroke for patients with certain co-morbidities.

### 6.1. Stroke type

Although the risk factors for ischemic and hemorrhagic stroke are similar, there are some differences. For example, hypertension and in particular high diastolic blood pressure are stronger risk factors for hemorrhagic stroke. Similarly some populations are more susceptible to one type of stroke over another, with those from developing countries more at risk for a hemorrhagic stroke (Zhang et al., 2005; Boehme et al., 2017). Thus, in some cases, models are created specifically for one type of stroke or the other in order to focus on the differences in these risk factors. While any risk model can provide insight on question 2 (b) i. from the introduction regarding risk factors that are strongly associated with stroke, the models that are specific to ischemic or hemorrhagic stroke answer question 2 (b) ii. and can help identify risk factors for different types of stroke. Although models for ischemic stroke are more common, in some cases the population being modeled requires a model for hemorrhagic stroke as well. For example, Zhang et al. (2005) create separate models for ischemic and hemorrhagic stroke specifically for a Chinese cohort. The authors advocate for specific models for the Chinese population because, while in Western countries CHD is more prevalent than stroke and the majority of strokes are ischemic strokes, in China stroke is more prevalent than CHD and both types of stroke are equally likely to occur. Using a Cox hazard model, the authors find a slightly different set of parameters for ischemic stroke vs. hemorrhagic stroke. The find that smoking has a significant impact on the risk of ischemic stroke but not hemorrhagic stroke and they use both systolic and diastolic blood pressure as risk factors for hemorrhagic stroke risk prediction, but only systolic blood pressure for ischemic stroke risk prediction. These results emphasize the importance of the authors creating two separate models, as certain risk factors contribute more to the overall risk of one type of stroke or the other. In analysis from the Eurostroke project, a number of risk factors, such as total and HDL cholesterol levels (Bots et al., 2002b), levels of fibrinogen (Bots et al., 2002a) and γ-Glutamyltransferase levels, are looked at for the separate contributions to ischemic and hemorrhagic stroke. While the analysis showed that levels of fibrinogen were a predictor for any kind of stroke, they found that levels of γ-Glutamyltransferase, which can be used as a marker for alcohol consumption, is a predictor for both types of stroke, but a stronger predictor for hemorrhagic stroke.

### 6.2. Co-morbidities

There are a number of conditions, or co-morbidites, that an individual might have that will make them more likely to suffer a stroke event. For these individuals it can be helpful to identify other risk factors that lead to an even higher increased risk of stroke. Identifying such factors, which might not have as big of an impact on the risk of the general population, might help to prevent stroke in those with the co-morbidity. As atrial fibrillation is an important risk factor for stroke, models have been developed to assess other risk factors for stroke, such as renal impairment, in population with atrial fibrillation (Banerjee et al., 2013). Other models created focus on the risk of diabetes for stroke patients (Banerjee et al., 2012) and the risk of pre-eclampsia in pregnant women (Brown et al., 2006). These studies help to show the increased risk that these co-morbidities can cause.

## 7. Using the risk characteristics to determine model type

While it is useful to understand the different types of risk and models being used in the field, understanding how the different categories of models are related to each other is an even more important aspect when creating a new risk model or understanding why a researcher made certain choices for their own model. We think that the questions outlined in the introduction can help guide a modeler in choosing model type and other aspects of their model. We repeat the questions here for ease of the reader:

1. Is the model for stroke specific risk or general CVD risk?
2. Is the study predicting stroke/CVD or looking at risk factors?
  (a) If the study is predicting stroke/CVD
    i. What is the probability (or risk) of an individual having a stroke/CVD within a preset time window (e.g., the next 10 years)?
    ii. What is the probability (or risk) of an individual having a stroke/CVD within their lifetime?
    iii. Within a given time window when is a person likely to have a stroke/CVD?
  (b) If the study is looking at risk factors
    i. What are the factors that have a strong association with stroke/CVD (i.e., what are the risk factors of stroke/CVD)?
    ii. Do different factors have a stronger association with a certain type of stroke/CVD (ischemic vs hemorrhagic)?
    iii. Are there risk factors that are more important for individuals with a co-morbidity?

**Figure 2:**
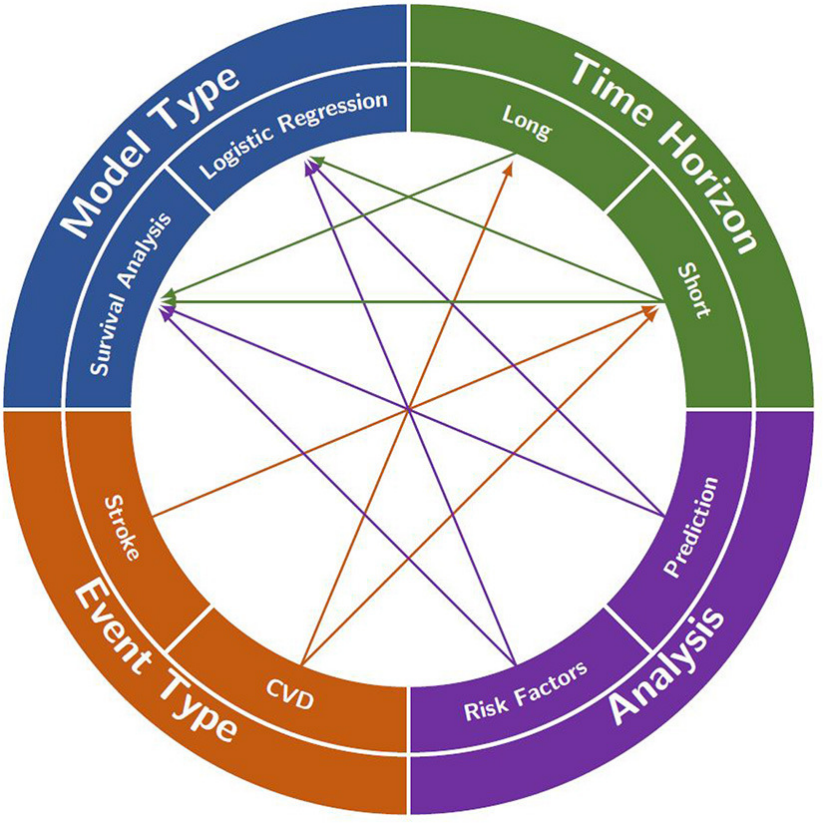
A visualization of the interconnections between analysis focus, event type, time horizon and model type that are often found in the literature. Each of the four characteristics is split into two main decisions to be made when creating a model (e.g., long- or short-time horizon). A decision in each category will often help to decide on one of the other categories (e.g., deciding to model stroke in event type will likely lead to the use of a short time horizon). However, in some cases, the decisions do not restrict any other categories (e.g., deciding to look at risk factors in the analysis does not restrict the model type).

Based on these questions, in this section we will outline the relationships between the event type, type of analysis, time horizon and how the decisions on those can help to choose the appropriate model to use and other aspects of the model. Table 1 at the end of the section is designed to help the reader understand how the different characteristics of the models fit together and what combinations of these characteristics exist in the literature as well as which of the questions the models answer. Figure 2 provides a visualization of this interconnectedness between characteristics of the models. While there are some sections below that suggest using one type of model over another to answer a certain question, for example survival analysis to model lifetime stroke risk, with the exception of having to use survival analysis if you are interested in the time to the stroke, the choice of model is largely a decision that comes down to the available data and the preference of the modeler. There are times when time to event data is not available, it is only recorded if an individual had a stroke in a given time period and not when in that time period, and thus survival analysis would be impossible. Additionally, in some scenarios one model might prove to produce better predictions than the other. In the below sections we aim to aid researchers in better understanding the choice between model types.

**Table 1:** Classification of risk models by a number of factors that define the model with examples of models that fit into each category.

**Analysis**	**Model type**	**Risk type**	**Event type**	**Time horizon**	**Example**
**2 (a) Prediction**	
i. Risk in a time window	Regression	Absolute/Odds Ratios	CVD	Short	Kannel et al., 1976
ii. Lifetime Risk	Survival Analysis	Absolute/Hazard Ratios	CVD	Long	Lloyd-Jones et al., 2006
iii. Time to Stroke	Survival Analysis	Hazard Ratios	Stroke	Short	Jee et al., 2008
**2 (b) Risk Factors**	
i. Stroke	Regression	Odds Ratios	Stroke	Short	Bots et al., 2002b
ii. Types of Stroke	Survival Analysis	Hazard Ratios	Ischemic/Hemorrhagic	Short	Zhang et al., 2005
iii. Co-morbidity	Regression	Odds Ratios	Ischemic Stroke	Short	Brown et al., 2006

### 7.1. Event type

As discussed in the introduction, one of the initial questions that should be asked when starting a risk modeling project is *Is the model for stroke specific risk or general CVD risk?* The risk factors for stroke and general CVD are often similar but the contributions for each risk factor to an individual's risk might vary between stroke type or between stroke and general CVD, as shown in Zhang et al. (2005). Because the contributions of the risk factors vary, it is important for the modeler to decide if they want to model specific stroke risk or more general CVD risk. The similarities in risk factors, however, mean that competing risks can be a problem when modeling stroke specifically. If someone has a high risk for a stroke they also will likely have a high risk for other CVD events. Thus, if the time horizon of the study is long the competing risk between stroke and other CVD events can lead to high levels of censoring, where people leave the data set before the end of the study due to another event, in this case a non-stroke CVD event. Therefore, choosing the event type can help decide the necessary time horizon for the model. As discussed in Section 4.1 there are a number of possible time horizons that can be chosen when predicting primary stroke risk, but these can be classified into short term (often 5–10 years) and long term or lifetime risk. If the model is predicting stroke or a specific sub type of stroke than the time horizon of the model should be a short term model. The shorter length of the prediction period and the study means that the competing risks and censoring will have less impact. Choosing a more general cardiovascular disease model does not limit the time horizon with models for both short and long term cardiovascular risk. Although there can still be censoring in the data for a general cardiovascular disease model, individuals may be lost to follow up or die due to non cardiovascular disease events, the competing risks between stroke types and other CVD events do not impact the model as the model is predicting a general CVD risk that encompasses all CVD events. Although choosing the event type for the model does not directly choose the type of model to use, we will discuss in later sections how the selection of time horizon from the event type can help to select a model type.

### 7.2. Type of analysis

The previous sections discuss how the answer to the first question on the event type can narrow down the choice of time horizon which will help to select a model type. The answer to the second question: *Is the study predicting stroke/CVD or looking at risk factors?*, can also help to select a model type. If the study is predicting stroke or general CVD then the modeler can look to the questions 2 (a) i., 2 (a) ii. and 2 (a) iii. to help determine the model type. If the model is focused on predicting if an event occurs during a time window question 2 (a) i. or 2 (a) ii as opposed to when in the time window a stroke occurs, question 2 (a) iii., than logistic regression can be used. This is because logistic regression provides the odds ratios for if an event occurred at all within the time window but not when in that time window the event occurred. Alternatively if the aim of the model is to take into account the time to event, question 2 (a) iii., then survival analysis should be used as survival analysis takes into account not only if the event occurred but when the event occurred.

If the studying aims to predict risk factors instead of stroke or CVD then the study will aim to answer one or more of questions 2 (b) i., 2 (b) ii. or 2 (b) iii. and is to look at associations between risk factors and stroke (what are risk factors, do risk factors differ by stroke type, do risk factors differ by co-morbidity by age). If this is the case than odds ratios are often chosen as the appropriate risk for the model and the model should be a logistic regression model. While odds ratios are often chosen in this case, the hazard ratios produced from survival analysis are also a measure of association and can be used to answer questions 2 (b) i., 2 (b) ii. or 2 (b) iii. The choice between logistic regression and survival analysis here can be a modeler's choice if they want to include time to event in their analysis of risk factors in which case survival analysis should be used over logistic regression. Additionally, the model type can be determined by the time horizon chosen (discussed in the next section).

### 7.3. Time horizon

As discussed in the previous sections, the answer to the question *Is the model for stroke specific risk or general CVD risk?*, can help to determine the time horizon but not the model type and while the answer to the question *Is the study predicting stroke/CVD or looking at risk factors?* might help to decide the model type, if the modeler is predicting risk factors, there might still be a question on what type of model to use. However, once the time horizon is known this can help to determine both the type of analysis being done and the type of model that should be used. A model that aims to predict long-term or lifetime risk or analyze long-term CVD risk factors, will typically result in a survival analysis model. This is because with lifetime risk it can be useful to understand not only if but when a stroke will occur. Additionally, survival analysis is known to better handle the censoring that will likely occur in a data set used to model long-term risk where individuals are often lost to follow up. Thus, if one is aiming to predict a long term or lifetime risk of CVD in a population, than they should choose an appropriate survival analysis technique.

Choosing short term risk does little to narrow down choices of model as either regression or survival analysis have been commonly used for short term risk prediction thus criteria other than time horizon will help to determine what type of model is used. Thus, while choosing to model lifetime CVD risk should result in creating a survival analysis model, short term models (either regression or survival analysis) can be created for general CVD, general stroke or specific stroke types. If short term stroke or CVD risk is being predicted, then the answers to questions 2 (a) i., 2 (a) ii., and 2 (a) iii. can help to determine model type as discussed in the previous section. If analysis aims to look at short term risk factors, then the modeler can determine if time to event should be included in the risk factors in which case survival analysis can be used if not logistic regression should be used.

## 8. Discussion

Having models that can accurately and reliably predict the risk of stroke is important in being able to identify individuals who are at the most risk for a stroke and to help mitigate their risk. Risk, however, is a concept that is often misunderstood and can mean a number of different things. Here we have reviewed the literature surrounding risk and risk models for primary stroke prevention and have broken them down into a number of different characteristics: the type of risk, the event type, the time horizon and the model type. These characteristics are interdependent and in some cases knowing one can help make the decisions about other parts of the model. For example if a model is aiming to predict lifetime risk it should be a model for absolute risk and should ideally predict cardiovascular disease, instead of more specifically stroke, to help account for competing risk. This classification can help those who are aiming to build a new risk model but can also help those who are researching risk models for prevention in gaining a better understanding of the different factors that should be considered when evaluating a risk model.

In our review, we have found that there has been much work done on predicting an individual's risk of stroke or cardiovascular disease, but there are still areas that can be further improved upon. Although there has been more of an emphasis on lifetime risk of stroke or cardiovascular disease in recent years with the addition of the recommendation to look at lifetime risk in the European stroke guidelines, the majority of the existing models still predict short-term risk. The short-term risk is helpful in identifying those who are in the most need of immediate risk reduction, however, a lifetime risk model might have identified such individuals earlier. Short-term models often give lower risk to certain groups such as younger individuals and in particular younger women. A lifetime model could help to identify risk factors that need to be lowered before they become an even larger problem and increase short-term risk. One approach to balancing short-term risk with the lifetime risk could be to present both measures to the clinician and patient as this could allow for more informed decision making. Alternatively, models by age could be created that predict over a longer time horizon for younger age groups and shorter time horizons for older age groups.

Currently, the majority of risk models used in stroke guidelines or in practice are created using either logistic regression and more recently survival analysis methods using a form of Cox regression. As such, in this review, we have not included models beyond logistic regression or survival analysis. While these models have proved to be robust, alternative methods such as neural networks, naive bayes, random forest, or SVM might have better predictive power, and in fact in some studies have been shown to increase predictive performance (Chun et al., 2021; Lip et al., 2022). While we feel that the questions and classification will likely apply to other types of models including neural networks or tree-based methods, a limitation of this review is that we do not consider these other model types in our risk concepts review and thus a similar review might be necessary to fully understand if there are different characteristics that need to be considered in a machine learning model beyond the type of risk, the event type, the time horizon and the model type. However, in reviewing different machine learning models for stroke risk prediction we have found that they focus on the performance of different models and do not provide information on the type of risk being modeled. Although exploring new techniques to improve risk prediction is an important task, it is also equally important for those new techniques to be presented in a way that is clinically meaningful. If a relative risk is misinterpreted as an absolute risk or an odds ratio as a relative risk, this could lead to incorrect decisions in terms of treatment that might have real consequences for a patient. Thus, for machine learning models to become best practice in the field of stroke risk prediction they should not only present model performance but also the risk concepts that are considered in their modeling. The set of questions we propose can be used to guide machine learning researchers to better define their models and place them in the stroke risk prediction literature. Additionally, it is important to consider the explainability of the model if it is to be used in a clinical setting as both the clinician and the patient will need to have a level of confidence in the model and its predictions in order for them to trust it. Even though a more complicated model might have better predictive power, than for example a logistic regression model, if it is a black box model it might not allow for this confidence. While explainable and interpretable AI have been a recent focus within the machine learning field, there is still evidence to show that clinicians and patients distrust in machine learning methods prevents greater uptake (Elish, 2018; Mpanya et al., 2021; Joshi et al., 2022).

Continuing with explainability it is essential to understand the type of risk that is being presented in the results of the model and how a patient and a clinician might interpret that risk. Multiple studies have shown that risk is easily misunderstood by both groups. While its claimed that absolute risk is more clinically meaningful, studies have shown that individuals are more likely to be responsive to relative risk. Thus creating a model that produces relative risk might be more beneficial if the aim is to get the patients to take the risk more seriously and reduce their known risk factors. This review aims to help readers better understand the difference between types of risk in the context of risk models.

Additionally, while we focus on models for primary stroke risk in this paper we feel that the same classification and recommendations would apply to a secondary stroke model. Further work could be done on reviewing and classifying the literature for secondary stroke models and comparing these models to primary stroke models to determine if there are key differences between them. Similar models to those described here are also used in stroke classification and prognostics. A review of the literature related to modeling post stroke outcomes could produce a similar classification to help better understand the models used there.

## 9. Conclusion

We have reviewed the literature on primary stroke risk prediction models and identified a number of systematic reviews of models designed to predict an individual's risk of primary stroke. However, there is no literature review that served as both a review of the literature and a guide for readers and modelers to better understand and interpret existing models. We have tried to fill this gap with this review that takes a novel approach of using the different characteristics of the modeling studies such as time horizon or event type to guide readers in understanding the choices made by modelers in their study. Additionally, we have proposed a checklist of questions that relate to these model characteristics that should be considered when creating a model. Although we only consider models for primary or initial stroke risk in this review, the methods used here, analyzing the literature, determining different characteristics that can be used to classify the models, finding the inter-dependencies between the characteristics and using those inter-dependencies to create a set of questions to guide future modeling can be applied to models for other events. In creating such reviews and classifications for different modeling fields, the fields will hopefully become more understandable and accessible to new modelers and researchers.

## Author contributions

EH and JK: conceptualization and methodology. EH: writing—original draft preparation. JK: writing—review and editing and supervision. Both authors have read and agreed to the published version of the manuscript.

## Funding

This project received funding from the EU's Horizon 2020 Research and Innovation Programme under grant agreement No. 777107, and by the ADAPT Centre for Digital Content Technology funded under the SFI Research Centres Programme (Grant No. 13/RC/2106_P2) and co-funded under the European Regional Development Funds.

## Conflict of interest

The authors declare that the research was conducted in the absence of any commercial or financial relationships that could be construed as a potential conflict of interest.

## Publisher's note

All claims expressed in this article are solely those of the authors and do not necessarily represent those of their affiliated organizations, or those of the publisher, the editors and the reviewers. Any product that may be evaluated in this article, or claim that may be made by its manufacturer, is not guaranteed or endorsed by the publisher.

## Footnotes

^1^Competing risks occur when there are at least two ways that a “failure” can occur (Kleinbaum and Klein, 2012). For example, in a longitudinal study looking at stroke risk factors, an individual could have a stroke or they could die from another cause prior to having a stroke.

^2^Censoring occurs when some but not all information about survival time is known. There are two types of censoring: Right censoring is when true survival time is greater than observed survival time in the study. It can occur because the event does not occur in the time window or a person is lost to follow-up or withdraws from the study. Left censoring is when the true survival time is less than the observed survival time and might occur when the exposure time is unknown (Kleinbaum and Klein, 2012).
